# Circulating Levels of Hydrogen Sulfide (H_2_S) in Patients with Age-Related Diseases: A Systematic Review and Meta-Analysis

**DOI:** 10.3390/biom13071023

**Published:** 2023-06-21

**Authors:** Eugenia Piragine, Marco Andrea Malanima, Ersilia Lucenteforte, Alma Martelli, Vincenzo Calderone

**Affiliations:** 1 Department of Pharmacy, University of Pisa, 56126 Pisa, Italy; eugenia.piragine@unipi.it (E.P.); vincenzo.calderone@unipi.it (V.C.); 2 Department of Clinical and Experimental Medicine, University of Pisa, 56126 Pisa, Italy; m.malanima@studenti.unipi.it (M.A.M.); ersilia.lucenteforte@unipi.it (E.L.)

**Keywords:** hydrogen sulfide, aging, age-related diseases, hypertension, type 2 diabetes, kidney disease, systematic review, meta-analysis

## Abstract

Hydrogen sulfide (H_2_S) is an endogenous gasotransmitter that promotes multiple biological effects in many organs and tissues. An imbalanced biosynthesis of H_2_S has been observed in animal models of age-related pathological conditions. However, the results from human studies are inconsistent. We performed a systematic review with meta-analysis of studies searched in Medline, Embase, Scopus, and CENTRAL databases. We included observational studies on patients with age-related diseases showing levels of H_2_S in blood, plasma, or serum. All the analyses were carried out with R software. 31 studies were included in the systematic review and 21 in the meta-analysis. The circulating levels of H_2_S were significantly reduced in patients with progressive, chronic, and degenerative diseases compared with healthy people (standardized mean difference, SMD: −1.25; 95% confidence interval, CI: −1.98; −0.52). When we stratified results by type of disorder, we observed a significant reduction in circulating levels of H_2_S in patients with vascular disease (e.g., hypertension) (SMD: −1.32; 95% CI: −2.43; −0.22) or kidney disease (SMD: −2.24; 95% CI: −4.40; −0.08) compared with the control group. These results could support the potential use of compounds targeting the “H_2_S system” to slow down the progression of many diseases in the elderly.

## 1. Introduction

Hydrogen sulfide (H_2_S) is a gaseous molecule with the typical smell of rotten eggs, known only as a toxic agent until 1996, when Abe and Kimura described for the first time its biosynthesis in mammals [1]. Together with carbon monoxide (CO) and nitric oxide (NO), H_2_S is now recognized as the third endogenous gasotransmitter, which exhibits a plethora of beneficial effects in many organs and tissues [2,3]. The biosynthesis of H_2_S can start directly from the amino acid L-Cysteine or derive from the condensation between L-Cysteine and homocysteine that occurs in the transsulfuration pathway [4]. Under physiological conditions, the endogenous production of H_2_S mainly results from the pyridoxal 5′-phosphate-dependent enzymatic activity of cystathionine β-synthase (CBS) and cystathionine γ-lyase (CSE), which are constitutively expressed in several systems with few differences in their localization. CSE is mainly present in the cardiovascular (CV) system, while CBS is expressed in the central nervous system (CNS), kidney, gut, liver, and pancreas. However, it is currently accepted that the localization of CBS and CSE enzymes is not so stringent, as CBS participates in the production of H_2_S in the CV system and vice versa [5]. The cysteine aminotransferase (CAT) and 3-mercaptopyruvate sulfurtransferase (3-MST) enzymes also take part in the biosynthesis of H_2_S in mammals [6] and contribute to the maintenance of physiological concentrations of the gasotransmitter in the blood [7,8]. Finally, a smaller amount of H_2_S derives from the sulfate-reducing activity of the intestinal microbiota [9].

Once produced, the small and lipophilic gaseous molecule H_2_S can rapidly cross cell membranes and act on many sarcolemmal and intracellular targets to produce multiple effects, from vasorelaxant to antioxidant and anti-inflammatory ones [10,11,12]. The precise mechanism of action of H_2_S has been described and consists mainly, but not exclusively, in the promotion of S-sulfhydration (or S-persulfidation) reactions on thiol residues of proteins [13,14]. This post-translational modification leads to changes in the structure and function of target proteins, thus modulating their activity [13]. For instance, H_2_S promotes the relaxation of blood vessels mainly via activation of ATP-sensitive potassium channels (K_ATP_) [10] and voltage-gated potassium channels (Kv7) [15], in part through S-sulfhydration reactions [16,17,18]. Moreover, via S-sulfhydration of cysteine residues, H_2_S protects cells from oxidative stress by activating the antioxidant Keap1/Nrf2/ARE pathway [19] and exerts anti-inflammatory effects by inhibiting the nuclear factor kappa-light-chain-enhancer of activated B cells (NF-κB) [20]. Therefore, both natural and synthetic compounds able to slowly release H_2_S (i.e., H_2_S-donors) have been recently proposed as potential candidates for the prevention and treatment of many CV and non-CV diseases characterized by chronic oxidative stress and sub-clinic inflammation, such as hypertension, cardiomyopathy, atherosclerosis, and type 2 diabetes (T2D) [21,22,23,24,25,26].

In this regard, the discovery of new therapeutic options able to prevent the onset of age-related diseases and slow their progression represents a major challenge nowadays since population aging is becoming a crucial issue in modern societies due to its massive impact on public health expenditure [27]. Of note, an impaired endogenous production of H_2_S has been observed in many animal models of age-related disorders (i.e., hypertension, T2D, atherosclerosis, etc.) [28,29,30]. The recent hypothesis that H_2_S has a role in the aging process further supports the use of H_2_S donors in the pharmacological treatment of age-related diseases to counteract the “oxi-inflamm-aging” process and reconstitute “H_2_S homeostasis”. However, a critical summary of the literature is still missing, and the clinical data are poor and quite controversial. For instance, as well as for the “king” of gasotransmitters NO, the endogenous production of H_2_S is reported to be enhanced under sudden inflammatory states or acute exacerbations of chronic diseases, probably to compensate for the endothelial damage and counteract the massive production of pro-inflammatory mediators that occurs after acute stimuli [31,32,33]. As concerns chronic age-related disorders, instead, many studies reported a marked decrease in H_2_S levels or expression of H_2_S-producing enzymes in patients with diabetes [23,34], hypertension [35], chronic hemodialysis (CHD), and chronic kidney disease (CKD) [36], while other studies showed an increased biosynthesis of H_2_S in aged people with disease [37].

A comprehensive overview of circulating levels of H_2_S could clarify the potential role of the gasotransmitter in the most common age-related diseases. This could support the pharmacological modulation of the endogenous “H_2_S system” (e.g., with H_2_S biosynthesis activators/inhibitors or H_2_S donors) to slow down the aging process and restore the physiological levels of the endogenous gasotransmitter in patients with disease, opening a new scenario in the management of aging. Furthermore, if a positive association between changes in the biosynthesis of H_2_S and the onset/progression of age-related disorders is confirmed, circulating levels of H_2_S might serve as a new biomarker for several diseases.

The aim of this systematic review and meta-analysis is to summarize evidence from papers investigating circulating levels of H_2_S in patients affected by the most common age-related disorders.

## 2. Materials and Methods

The protocol has been registered in the PROSPERO database (CRD42023405958). The systematic review and meta-analysis have been performed following the preferred reporting items for systematic reviews and meta-analysis (PRISMA) guidelines.

### 2.1. Search Strategy and Study Selection

Medline (via Pubmed), Scopus, Embase, and CENTRAL (via the Cochrane Library) databases have been used for searching studies published until 7 December 2022. The search strategy was generated by combining three terms with the Boolean operator “AND”. Hydrogen sulfide represented the first term, a biological sample (blood, plasma, or serum) the second term, and the pathological condition of interest (age-related disorders) the third term (Supplementary Material S1).

Two authors, E.P. and M.A.M., screened titles and abstracts independently. Based on the inclusion and exclusion criteria, papers were classified as non-relevant or potentially eligible. Any disagreement was discussed with another author (E.L.).

The full text of the potentially eligible articles was then retrieved. Whenever possible, unavailable full texts were directly requested from the first author of the study. Moreover, two authors (E.P. and M.A.M.) checked the full texts and excluded studies that did not meet the predefined inclusion criteria.

The selection process was managed using the bibliographic management software Mendeley Desktop (v1.19.6).

### 2.2. Inclusion and Exclusion Criteria

We included studies on patients with the most common age-related disorders (hypertension, T2D, cancer, etc.) [38,39], without restrictions of gender, age, or presence of comorbidities. The primary variable of interest was the circulating levels of H_2_S measured in blood, plasma, or serum. We excluded records reporting concentrations of H_2_S in organs and tissues, as they are not directly comparable to circulating levels of H_2_S. We included comparative observational studies (cohort, case-control, and cross-sectional). Studies not written in English, abstracts/posters, letters to editors, reviews, clinical trials, and case reports were also excluded.

### 2.3. Data Extraction

We extracted the following information: study design; number and general characteristics of patients, such as age and gender; list of the exclusion criteria; matching methods, if described; biological sample used; analytical technique employed; description of sample collection; circulating levels of H_2_S in patients with and without disease. When different stages of disease were considered, the mean concentration of H_2_S was extracted or calculated.

The data collection was carried out independently by two authors, E.P. and M.A.M., using the spreadsheet software Microsoft Excel (version 2102 build 13801.20864). Any discrepancies were discussed with a third reviewer (E.L.).

### 2.4. Quality Assessment

The methodological quality of the included studies was assessed independently by two authors (E.P. and M.A.M.) with a modified version of the Joanna Briggs Institute (JBI) Critical Appraisal Checklist for case-control studies or cross-sectional studies. The checklist for case-control studies is composed of 10 domains evaluating the comparability between groups, the appropriateness of the matching method, the clarity of inclusion/exclusion criteria, the description of study subjects and settings, the size of experimental groups, the close representation of the target/reference population under investigation, the identification of confounding factors and/or strategies to deal with confounding factors, the validity and reliability of the H_2_S measurement method, and the statistical analysis used. Each domain was classified as having a high risk of bias (score = 0) or a low risk of bias (score = 1). For case-control studies, the total score ranged from 0 to 10. Studies were classified as at high risk of bias for a total score of 0–5, at moderate risk of bias for a total score of 6–8, and at low risk of bias for a total score of 9–10.

For cross-sectional studies, the comparability between groups and the appropriateness of the matching method were not considered in the JBI Critical Appraisal Checklist. Therefore, the maximum total score for cross-sectional studies was 8. Studies were classified as having a high risk of bias for a total score of 0–4, a moderate risk of bias for a total score of 5–6, and a low risk of bias for a total score of 7–8.

### 2.5. Statistical Analysis

Studies reporting circulating levels of H_2_S in patients with disease and healthy controls were included. We used concentration values calculated as mean ± standard deviation (SD). For studies reporting the standard error of the mean (SEM), SD was obtained by multiplying the SEM by the square root of the number of patients per group. If results of the primary studies were not shown as mean with SEM or SD (i.e., for studying reporting values as median and interquartile range), we estimated means and SD according to the equations reported by Hozo et al. [40]. We performed meta-analyses of standardized mean differences (SMD) using random effect models. Pooled SMD were considered significant if the reported 95% confidence intervals did not include 0. The inverse variance method was used to obtain study-specific weights, while the restricted maximum-likelihood (REML) estimate was used for estimating tau squared (τ ^2^). Higgins I^2^ statistic was used to investigate heterogeneity. An overall meta-analysis was performed, including all patients, to evaluate the difference in circulating H_2_S levels between patients with disease and healthy subjects. We also performed meta-analyses stratified by etiopathogenesis and/or clinical features of the included diseases and by the most represented chronic age-related diseases (i.e., vascular diseases, T2D, and CKD). For studies reporting more than one disease group, we aggregated their means and SD weightings by sample size. For T2D and CKD, sensitivity analyses were also performed to evaluate the difference between SMD resulting from aggregated and non-aggregated diseases. A stratified meta-analysis according to the risk of bias was performed. For all meta-analyses, influence analyses were also performed. All the analyses were carried out with R software version 4.2.2.

## 3. Results

### 3.1. Systematic Review

**Figure 1:**
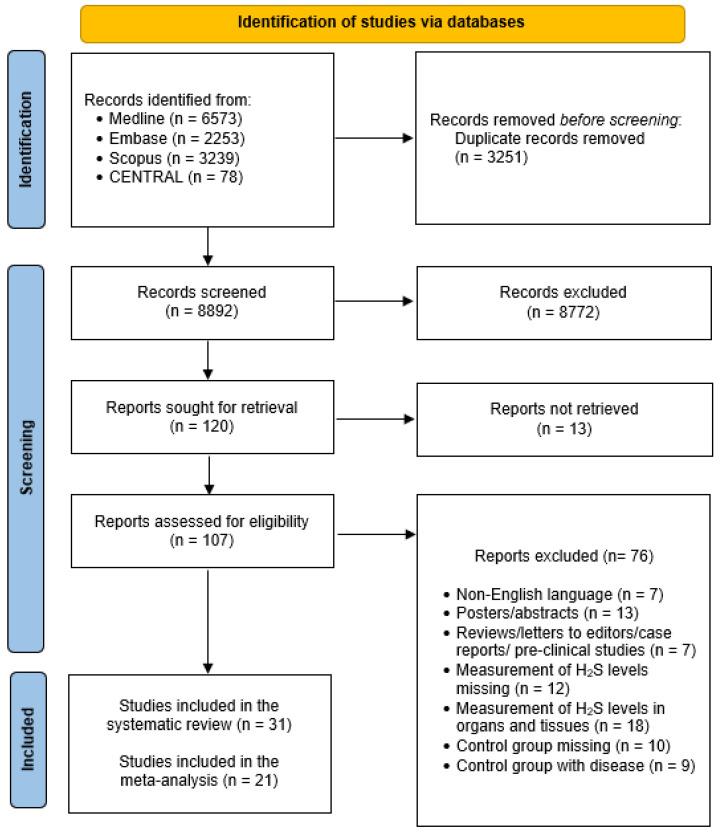
Flow chart of the search.

Records identified were 6573 through Medline searching, 2253 through Embase searching, 3239 through Scopus searching, and 78 through CENTRAL searching. After removal of duplicates, 8892 titles and abstracts were screened, and 107 full texts were assessed for eligibility. According to the inclusion criteria, 76 studies were excluded, and the qualitative synthesis was conducted on 31 records [37,41,42,43,44,45,46,47,48,49,50,51,52,53,54,55,56,57,58,59,60,61,62,63,64,65,66,67,68,69,70]. Of them, 10 studies showed circulating levels of H_2_S exclusively in graphical form. Therefore, meta-analysis was performed on 21 studies [42,43,46,47,48,53,54,55,56,57,58,59,61,62,63,64,65,67,68,69,70] (Figure 1).

The results of the systematic review are shown in detail in Table S1 and summarized in Table 1. Twenty-four records included in the qualitative synthesis were case-control studies [37,41,42,44,45,46,48,49,51,52,53,54,56,57,58,60,61,62,64,65,66,67,68,70], while seven were cross-sectional studies [43,47,50,55,59,63,69]. Most papers reported circulating levels of H_2_S in patients with chronic age-related diseases, except for the two records evaluating concentrations of H_2_S in patients with acute myocardial infarction (AMI) [41,42]. In these studies, biological samples (serum or plasma) were collected 1–10 h after the clinical manifestation of AMI (chest pain).

The most represented disorders were T2D [43,49,51,52,61,62,64,67], kidney disease (CKD/CHD) [48,53,54,58,66], kidney disease plus T2D [48,54], hypertension [37,46,57,65,69], vascular disease [47,56,59], and cancer [37,68]. Patients with disease had a mean age of 54.2 years, and 79.4% were men. In the control group, the mean age was 48.2 years, and 68.6% were men. The control group consisted of healthy patients or, more generally, patients without the disease of interest. However, in some studies, patients in the control group and patients with diseases shared comorbidities. This apparent difference in age, gender, and baseline risk between patients with and without disease was considered in the items related to comparability between groups and the presence of confounding factors in the quality assessment tools (Section 3.2).

Most studies reported the concentration of H_2_S in plasma [37,42,45,47,48,49,53,54,55,56,57,58,59,60,62,64,65,66,67,69] or serum [41,43,44,46,50,63,70], except for three studies in which levels of H_2_S have been measured in the whole blood [51,52,68]. Several methods have been used to detect circulating levels of H_2_S, but the most prevalent were spectrophotometric methods (methylene blue) [43,47,48,51,52,53,55,57,58,62,64,65,67], sulfide-sensitive electrodes [44,49,54,63,66,70], and the use of fluorescent/luminescent probes [42,46,68]. Other techniques included the use of ELISA kits [37,41,50], high performance liquid chromatography (HPLC) [45,59], liquid chromatography coupled with mass spectrometry (LC-MS/MS) [61,69], gas chromatography (GC) [60], and the lead acetate method [56].

### 3.2. Risk of Bias Assessment

Table S2 shows the results of the risk of bias assessment of case-control studies. Fourteen out of twenty-four case-control studies included in the systematic review were endowed with a high risk of bias (score 0–5 in the JBI Critical Appraisal Checklist for case-control studies) [37,41,42,44,45,46,48,49,51,56,60,64,67,68], mainly for the absence of clearly stated inclusion and exclusion criteria, for the lack of heterogeneity of the studied population (which made patients with disease not representative of the “real-world” population for age, gender, or presence of comorbidities), for the use of an analytical technique with low validity and sensitivity, and for the presence of confounding factors that were not considered by the authors. Eight studies have been classified as having “moderate risk of bias” (scores between 6 and 8 in the JBI scale) [50,52,53,57,58,62,65,66,70], while two studies reached the highest scores (9 and 10), and they were considered studies with “low risk of bias” [54,61].

Table S3 shows the results of the risk of bias assessment of cross-sectional studies. One out of seven cross-sectional studies had a high risk of bias (score 0–4 in the JBI Critical Appraisal Checklist for cross-sectional studies) [69], mainly for the presence of confounding factors and the lack of information about predefined inclusion/exclusion criteria. Five studies were endowed with moderate risk of bias (score 5–7 in the JBI Checklist) [43,47,50,59,63], while one study was considered to have “low risk of bias” (score 8/8 in the JBI) [55].

### 3.3. Results of Synthesis

#### 3.3.1. Circulating Levels of H_2_S in Patients with Age-Related Diseases 

**Figure 2:**
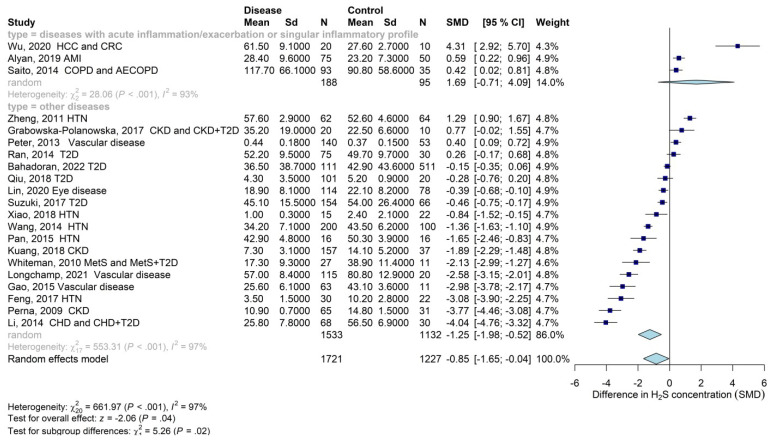
Forest plot with standardized mean difference (SMD) of circulating levels of H_2_S in patients with age-related diseases characterized by acute inflammation (e.g., AMI), acute exacerbations of disease (e.g., COPD), or a singular inflammatory profile (e.g., cancer), as well as chronic age-related diseases associated with a gradual decrease in organ and tissue functions and low-grade inflammation (other diseases) compared with subjects without disease (control group). Abbreviations: AE-COPD, acute exacerbation of chronic obstructive pulmonary disease; AMI, acute myocardial infarction; COPD, chronic obstructive pulmonary disease; CHD, chronic hemodialysis; CKD, chronic kidney disease; COPD, chronic obstructive pulmonary disease; CRC, colorectal cancer; CKD, chronic kidney disease; HCC, hepatic cancer; HTN, hypertension; MetS, metabolic syndrome; T2D, type 2 diabetes. References: [42,43,46,47,48,53,54,55,56,57,58,59,61,62,63,64,65,67,68,69,70].

The results of the overall meta-analysis (1721 patients with disease and 1227 healthy subjects) showed that circulating levels of H_2_S were significantly lower in patients with age-related diseases compared with the control group (SMD: −0.85; 95% CI: −1.65; −0.04) (Figure 2), with consistent heterogeneity (I^2^ = 97%). To identify a possible source of heterogeneity, we first grouped disorders by etiopathogenesis/clinical features.

All the diseases included in the overall analysis are characterized by inflammation. Some of them are associated with acute inflammation (e.g., AMI) or sudden inflammation due to exacerbations (e.g., COPD/AE-COPD) [71]. However, most diseases have low-grade and chronic inflammation (e.g., T2D, kidney disease, hypertension, etc.) [72,73,74]. The recently proposed “inflammatory classification system” [75] also states that the type and levels of cytokines can differ between various inflammatory states, suggesting that measurement of cytokine parameters could help determine the primary cause of inflammation. For instance, inflammation due to cancer can lead to the release of cytokines other than those induced by diabetes. Thus, we classified the diseases into two groups: those characterized by acute inflammation at onset/exacerbation or by a singular inflammatory profile (i.e., AMI, COPD, and cancer) and those associated with a gradual decrease in organ and tissue functions and low-grade inflammation (e.g., T2D, kidney disease, hypertension, etc.). In our analysis, the circulating levels of H_2_S were higher in patients with AMI, COPD, or cancer compared with healthy patients (SMD: 1.69; 95% CI: −0.71; 4.09) (Figure 2). On the contrary, patients with chronic age-related pathologies characterized by low-grade inflammation and a gradual decrease in organ and tissue functions showed a marked reduction in the levels of H_2_S (SMD: −1.25; 95% CI: −1.98; −0.52) (Figure 2). Of note, the difference between groups was significant (*p*-value = 0.02).

When we stratified studies by risk of bias, the results showed no significant difference between groups (*p*-value = 0.90) for studies on disorders characterized by a gradual decrease in organ and tissue functions and low-grade inflammation (Figure S1), as well as for the other studies (*p*-value = 0.29) (Figure S2). In all cases, the results of the influence analyses, performed by removing each study one by one, did not show a change in terms of the direction of the effect.

Studies not included in the meta-analysis showed a similar trend (Table S1). Those on patients with multiple myeloma [37], AMI [41], COPD/AE-COPD [44], and osteopenia/osteoporosis [50] reported an increase in circulating levels of H_2_S in subjects with disease compared with the control group, while studies on patients with T2D [51,52], diabetic cardiomyopathy [49], and CHD [66] showed a decrease in circulating levels of H_2_S. Two studies reported no change in circulating levels of the gasotransmitter in patients with heart failure [60] and Alzheimer’s disease and related dementias (ADRDs) [45].

#### 3.3.2. Circulating Levels of H_2_S in Patients with Diseases Characterized by a Gradual Decrease in Organ and Tissue Functions and Low-Grade Inflammation

**Figure 3:**
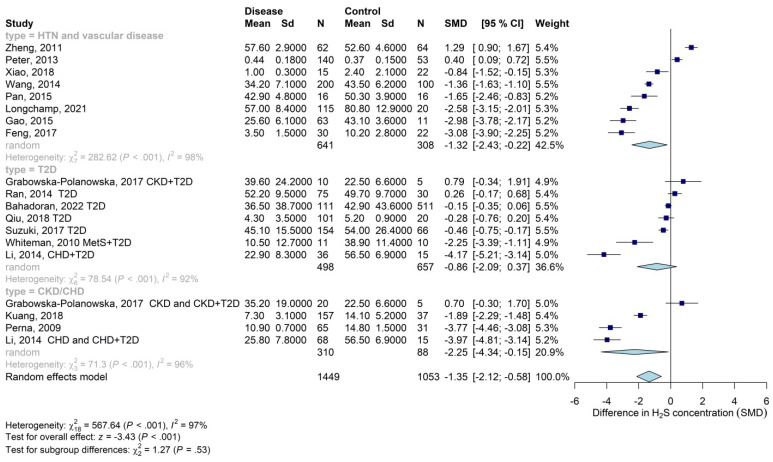
A forest plot with standardized mean difference (SMD) of circulating levels of H_2_S in patients with CVDs (hypertension and vascular disease) (first group), T2D (second group), or CKD/CHD (third group) compared with subjects without disease (control group). Abbreviations: CHD, chronic hemodialysis; CKD, chronic kidney disease; HTN, hypertension; MetS, metabolic syndrome; T2D, type 2 diabetes. Considered vascular diseases (when specified): angina, coronary artery disease, or peripheral artery disease. References: [43,46,47,48,53,54,56,57,58,59,61,62,64,65,67,69,70].

Given the high heterogeneity observed in the overall analysis, we performed a stratified analysis by specific type of disease. We considered the most represented age-related diseases in the included studies, i.e., CVDs, T2D, and CKD (Figure 3). There was no significant difference between subgroups (*p*-value = 0.53).

As concerns CVDs (hypertension or vascular diseases), the meta-analysis of eight studies showed that circulating levels of H_2_S were significantly lower in patients with CVDs compared with the control group (SMD: −1.32; 95% CI: −2.43; −0.22), with high heterogeneity (I^2^ = 98%).

The meta-analysis of four studies on patients with T2D and relative controls did not show a significant difference in circulating levels of H_2_S between patients with T2D and patients without T2D (SMD: −0.87; 95% CI: −2.17; 0.43), with high heterogeneity (I^2^ = 94%). Sensitivity analysis, including studies with only T2D patients and relative controls, confirmed a non-significant difference in SMD (data not shown).

The meta-analysis of four studies evaluating the circulating levels of H_2_S in patients with CKD or CHD showed that circulating levels of the gasotransmitter were significantly lower in patients with CKD or CHD compared with the control group (SMD: −2.24; 95% CI: −4.40; −0.08), with consistent heterogeneity (I^2^ = 97%). Sensitivity analysis, including studies on patients with only CKD or CHD (without comorbidities), confirmed a significant difference in SMD compared with the control group (data not shown).

## 4. Discussion

In recent decades, human life expectancy has progressively increased, especially in Western countries. As a major consequence, the incidence and prevalence of multiple age-related disorders (i.e., CVDs, T2D, and cancer) have grown exponentially, with a large impact on global health and healthcare costs. Therefore, the discovery of new biomarkers of aging, as well as compounds able to prevent the aging process, is a compelling need. Under physiological conditions, the gasotransmitter H_2_S plays a crucial role in the regulation of tissue homeostasis: it potentiates the endogenous antioxidant defense system, counteracts the inflammatory process, and slows down cellular senescence. In many pre-clinical studies, an age-dependent impairment in the biosynthesis of H_2_S has been described, but the results of clinical and observational studies are inconclusive. 

In this systematic review, we demonstrated that circulating levels of H_2_S significantly change in patients with the most common age-related disorders compared with healthy subjects. This evidence strengthens the results of animal studies and indicates that, in the elderly, the dysregulation of tissue homeostasis could be associated with an impaired biosynthesis of H_2_S, although a cause-and-effect relationship is still unclear. This tendency was confirmed in the meta-analysis of 21 studies, which showed a global reduction in plasma levels of H_2_S in older people with disease. However, the heterogeneity was consistent. When we stratified results by risk of bias, we did not find significant differences between groups, suggesting that the methodological quality of the included studies was not a source of heterogeneity. Conversely, the inclusion of different types of age-related disorders might have partially contributed to the high heterogeneity of the main analysis. To avoid misinterpretation of the results, we grouped age-dependent diseases by etiopathogenesis and/or clinical features and found significant differences between groups. 

One group included pathological conditions characterized by acute inflammation (e.g., AMI), acute exacerbations of disease (e.g., COPD), or a singular inflammatory profile (e.g., cancer), according to the “inflammatory classification system” [75]. Briefly, AMI is associated with acute, dynamic, and systemic inflammation, which is clinically detectable by measuring plasma levels of C-reactive protein (CRP) and other markers of inflammation [76,77,78,79]. Of note, the release of CRP has been positively correlated with recurrent AMI [76]. COPD, instead, is a singular disease characterized by chronic progression interrupted by acute phases of exacerbation (AE-COPD). These episodes are associated with acute and systemic inflammation, which in part is similar to that described for AMI [80,81]. Our results showed a trend toward increasing circulating levels of H_2_S in subjects with AMI and COPD. The release of H_2_S following a sudden inflammatory state, which occurs in patients with AMI or acute COPD, might be a compensatory mechanism to neutralize the massive injury induced by oxidative stress and rapid activation of pro-inflammatory pathways, as previously proposed [32,33,82,83]. In this regard, a recent meta-analysis of pre-clinical studies demonstrated the efficacy of H_2_S donors in counteracting post-ischemic events in the myocardium subjected to ischemia/reperfusion injury, concluding that post-conditioning the heart with exogenous sources of H_2_S may represent a possible therapeutic strategy to limit the infarct size and, subsequently, the cardiac damage [84]. Furthermore, many studies support our findings on increasing circulating levels of H_2_S in patients with cancer. Indeed, enhanced expression of CBS and CSE enzymes in human cancer cells has been widely reported. This leads to abnormal biosynthesis of H_2_S and positively correlates with worse clinical outcomes (e.g., enhanced tumor growth, angiogenesis, metastasis formation, and tumor cell resistance) [85,86,87,88,89]. The low number of studies included in our meta-analysis does not allow us to further discuss the possible role of H_2_S in the onset and progression of these age-related pathological conditions. However, our results strengthen the hypothesis that H_2_S could be considered a new biomarker for age-related disorders such as cancer and AMI.

The second group included diseases characterized by low-grade, subclinical inflammation and persistent oxidative stress (e.g., T2D, hypertension/vascular disease, and CKD/CHD), which represent the most common pathological conditions in the elderly. Our meta-analysis showed that circulating levels of H_2_S are significantly lower in patients with these age-dependent diseases compared with healthy controls, without any difference between groups.

However, given the high heterogeneity observed, we performed a subgroup analysis by type of disease (i.e., vascular disorders, CKD, and T2D). A significant reduction in the circulating concentration of the sulfur gasotransmitter has been detected in patients with chronic vascular diseases (i.e., hypertension and coronary artery disease). Accordingly, a deficient endogenous production of H_2_S has been previously demonstrated in pre-clinical models of hypertension [90,91]. Observational studies also confirmed that reduced circulating levels of H_2_S, which could result from an altered expression/activity of H_2_S-producing enzymes (i.e., CSE and CBS) or H_2_S-metabolizing enzymes (i.e., sulfide-quinone oxidoreductase, SQR; thiosulfate sulfurtransferase, TST; persulfide dioxygenase, ETHE-1) in the senescent tissues, might contribute to the onset and progression of vascular diseases [92]. For instance, low levels of the CBS gene due to epigenetic imbalance (i.e., CBS hypermethylation) enhanced the risk of hypertension in humans [93]. This epigenetic alteration leads to an imbalance in the transsulfuration pathway as it reduces the conversion of homocysteine into cysteine, with the subsequent development of hyperhomocysteinemia, a recognized risk factor for vascular diseases [94,95]. At the same time, the reduced expression of CBS leads to a deficient biosynthesis of H_2_S. Which of hyperhomocysteinemia, impaired biosynthesis of H_2_S, or both is directly involved in the onset and progression of vascular diseases is not well established, but many studies support the use of compounds targeting the “H_2_S-system” in the treatment of hypertension [24,96], mainly due to the antihypertensive effects of the gasotransmitter [97] and its emerging role in the regulation of epigenetic mechanisms [98]. In addition, the impaired biosynthesis of H_2_S in patients with hypertension confirms the potential use of the sulfur gasotransmitter as a biomarker of the disease. Of course, hyperhomocysteinemia could be a possible confounder associated with reduced circulating levels of H_2_S during aging. Other confounders, such as vascular calcification and vascular fibrosis, may also alter vascular homeostasis, leading to potential changes in endogenous H_2_S production. However, regardless of the cause of “H_2_S imbalance”, the hypothesis that many pathological conditions might lead to reduced levels of the gasotransmitter during aging suggests the role of H_2_S as a “final” and “common” biomarker of the aging process.

Our analysis also demonstrated that circulating levels of H_2_S are reduced in patients with kidney disease. Accordingly, a marked down-regulation of CBS, CSE, and MST in the kidney has been reported in many animal models [36,99], while treatment with H_2_S-donors restored physiological H_2_S levels and improved renal function in pre-clinical studies [36,100]. Of note, a gradual decline in circulating levels of H_2_S during the progression of kidney disease has been shown [53], further supporting the potential involvement of endogenous H_2_S in the pathogenesis of age-related disorders characterized by low-grade inflammation and oxidative stress, including kidney disease. On the contrary, the results of studies evaluating the circulating levels of H_2_S in patients with T2D were inconclusive. A previous meta-analysis showed increased plasma concentrations of the gasotransmitter NO in patients with T1D and T2D, probably due to the activation of inducible endothelial nitric oxide synthase (iNOS), which is overexpressed under inflammatory conditions [101]. Worthy to note, both H_2_S and H_2_S-donors have been described as inhibitors of iNOS expression and activity [102,103,104]. Hence, the enhanced production of NO in patients with T2D might, in part, result from the deficient biosynthesis of H_2_S, which has been reported in many animal models of diabetes [23] but not fully demonstrated in clinical studies. A progressive decrease in circulating levels of H_2_S during the progression of T2D has also been shown [43]. However, the possible role of H_2_S in patients with T2D must be confirmed to support the possible use of H_2_S signaling modulators in the prevention and treatment of T2D and its CV complications.

## 5. Conclusions

Age-related disorders represent an impressive threat for healthcare systems nowadays, and the discovery of novel biomarkers of aging and innovative therapeutic options may allow for better clinical management of the most common age-related diseases. This is the first meta-analysis to produce a comprehensive overview of the levels of H_2_S in patients with age-related disease. In this study, we demonstrated that the circulating concentration of the gasotransmitter H_2_S changes in patients with age-dependent disorders. Indeed, H_2_S levels appear higher under pathological conditions characterized by acute inflammation or a singular inflammatory profile and lower in patients with age-related disorders associated with the “oxi-inflammaging process” (i.e., hypertension and CKD). Our results, although preliminary, suggest that circulating levels of the gasotransmitter H_2_S may serve as a new biomarker of aging, with a potential clinical use to “prevent” changes in H_2_S biosynthesis and to follow the progression of age-related disorders. Moreover, our data could support the potential use of “H_2_S-system” modulators to slow down the aging process and the onset/progression of a wide range of disorders in the elderly.

This study has some limitations. First, H_2_S is biosynthesized by the catalytic activity of the enzymes of the transsulfuration pathway, which involves other sulfur species (i.e., homocysteine) whose potential role in the aging process cannot be excluded. Moreover, most of the studies included in our analysis were at moderate/high risk of bias, mainly due to the presence of potential confounding factors or the low sensibility and sensitivity of the H_2_S detection method used. For instance, approximately half of the studies included in our systematic review used the methylene blue method for measuring circulating levels of H_2_S in biological samples. We are aware that incorporating results from studies using this experimental technique may have introduced a possible source of bias into our analysis. We have considered this crucial aspect in the risk of bias assessment to partly overcome this limitation. Finally, we focused exclusively on the gasotransmitter H_2_S, as it is the “final effector” of multiple, pleiotropic biological effects, but many other sulfur species (e.g., polysulfides) might be used as a “proxy” of H_2_S levels. However, the current literature on the measurement of polysulfide levels in patients with disease is not related to age-related disorders. The identification of easily detectable H_2_S “derivatives” could be a possible future direction of this study to provide a complete panorama of the role of the endogenous sulfur species in patients with age-related disorders.

## Figures and Tables

**Table 1:** Summary of the characteristics of the studies included in the systematic review. List of abbreviations: CHD: chronic hemodialysis; CKD: chronic kidney disease; COPD: chronic obstructive pulmonary disease; T2D: type 2 diabetes. Legend: * gas chromatography (GC, one study); high performance liquid chromatography (HPLC, two studies); lead acetate (one study); liquid chromatography-tandem mass spectrometry (LC-MS/MS, two studies). ** acute exacerbation of chronic obstructive pulmonary disease/chronic obstructive pulmonary disease (AE-COPD/COPD, three study arms) [44,63]; acute myocardial infarction (two study arms) [41,42]; Alzheimer’s disease and related dementias (one study arm) [45]; heart failure (one study arm) [60]; ocular disease (ocular hypertension, normal tension glaucoma, primary open-angle glaucoma, three study arms) [55]; osteopenia/osteoporosis (one study arm) [50]; overweight (one study arm) [67].

Study Design (*n*. of Studies; *n*. of Patients/Controls)	Case Control: 24; 1581/690 Cross-Sectional: 7; 640/785
Mean age	Control: 48.2
(years)	Disease: 54.2
Gender	Control: 78.6
(male, %)	Disease: 79.4
Biological sample (*n*. of studies)	Plasma: 20
	Serum: 8
	Blood: 3
Measurement Method (*n*. of studies)	Spectrophotometric method: 13
	Sulfide-sensitive electrodes: 6
	ELISA kit: 3
	Fluorescent/luminescent probes: 3
	Others *: 6
Type of disease (*n*. of study arms; *n*. of patients/controls)	T2D: 7; 541/688
	T2D + proliferative retinopathy: 1; 25/25
	T2D + non-proliferative retinopathy: 1; 25/25
	T2D + cardiomyopathy: 1; 32/-
	CKD/CHD: 5; 328/138 CHD + T2D: 2; 42/40
	Hypertension: 5; 317/224
	Vascular disease: 3; 397/84
	Cancer: 3; 40/25
	Others **: 10; 474/327

## Data Availability

The data that support the findings of this study are available from the corresponding author upon reasonable request.

## Supplementary Materials

The following supporting information can be downloaded at: https://www.mdpi.com/article/10.3390/biom13071023/s1. S1: Search strategy; Table S1: Characteristics of the included studies, details of H_2_S level measurements and main results; Table S2: Risk of bias in case-control studies; Table S3: Risk of bias in cross-sectional studies; Figure S1: Figure S1. Forest plot with standardized mean differences (SMD) of circulating levels of H2S, stratified by risk of bias, in patients with chronic age-related diseases characterized by a gradual decrease in organ and tissue functions and low-grade inflammation compared with subjects without disease (control group); Figure S2: Forest plot with standardized mean differences (SMD) of circulating levels of H2S, stratified by risk of bias, in patients with age-related diseases characterized by acute inflammation (e.g., AMI), acute exacerbations of disease (e.g., COPD), or singular inflammatory profile (e.g., cancer) compared with subjects without disease (control group).

## References

[1] Abe K., Kimura H. (1996). The possible role of hydrogen sulfide as an endogenous neuromodulator. J. Neurosci..

[2] Wang R. (2010). Hydrogen sulfide: The third gasotransmitter in biology and medicine. Antioxid. Redox Signal..

[3] Citi V., Piragine E., Testai L., Breschi M.C., Calderone V., Martelli A. (2018). The Role of Hydrogen Sulfide and H2S-donors in Myocardial Protection Against Ischemia/Reperfusion Injury. Curr. Med. Chem..

[4] Sbodio J.I., Snyder S.H., Paul B.D. (2019). Regulators of the transsulfuration pathway. Br. J. Pharmacol..

[5] Cao X., Ding L., Xie Z.Z., Yang Y., Whiteman M., Moore P.K., Bian J.S. (2019). A Review of Hydrogen Sulfide Synthesis, Metabolism, and Measurement: Is Modulation of Hydrogen Sulfide a Novel Therapeutic for Cancer?. Antioxid. Redox Signal..

[6] Stipanuk M.H. (2004). Sulfur amino acid metabolism: Pathways for production and removal of homocysteine and cysteine. Annu. Rev. Nutr..

[7] Olson K.R. (2009). Is hydrogen sulfide a circulating "gasotransmitter" in vertebrate blood?. Biochim. Biophys. Acta.

[8] Whitfield N.L., Kreimier E.L., Verdial F.C., Skovgaard N., Olson K.R. (2008). Reappraisal of H2S/sulfide concentration in vertebrate blood and its potential significance in ischemic preconditioning and vascular signaling. Am. J. Physiol. Regul. Integr. Comp. Physiol..

[9] Blachier F., Andriamihaja M., Larraufie P., Ahn E., Lan A., Kim E. (2021). Production of hydrogen sulfide by the intestinal microbiota and epithelial cells and consequences for the colonic and rectal mucosa. Am. J. Physiol. Gastrointest. Liver Physiol..

[10] Zhao W., Zhang J., Lu Y., Wang R. (2001). The vasorelaxant effect of H_2_S as a novel endogenous gaseous K(ATP) channel opener. EMBO J..

[11] Chen T., Tian M., Han Y. (2020). Hydrogen sulfide: A multi-tasking signal molecule in the regulation of oxidative stress responses. J. Exp. Bot..

[12] Whiteman M., Winyard P.G. (2011). Hydrogen sulfide and inflammation: The good, the bad, the ugly and the promising. Expert Rev. Clin. Pharmacol..

[13] Ju Y., Fu M., Stokes E., Wu L., Yang G. (2017). H_2_S-Mediated Protein S-Sulfhydration: A Prediction for Its Formation and Regulation. Molecules.

[14] Mustafa A.K., Gadalla M.M., Sen N., Kim S., Mu W., Gazi S.K., Barrow R.K., Yang G., Wang R., Snyder S.H. (2009). H_2_S signals through protein S-sulfhydration. Sci. Signal..

[15] Martelli A., Testai L., Breschi M.C., Lawson K., McKay N.G., Miceli F., Taglialatela M., Calderone V. (2013). Vasorelaxation by hydrogen sulphide involves activation of Kv7 potassium channels. Pharm. Res..

[16] Jiang B., Tang G., Cao K., Wu L., Wang R. (2010). Molecular mechanism for H_2_S-induced activation of K(ATP) channels. Antioxid. Redox Signal..

[17] Kang M., Hashimoto A., Gade A., Akbarali H.I. (2015). Interaction between hydrogen sulfide-induced sulfhydration and tyrosine nitration in the KATP channel complex. Am. J. Physiol. Gastrointest. Liver Physiol..

[18] Piragine E., Citi V., Lawson K., Calderone V., Martelli A. (2022). Regulation of blood pressure by natural sulfur compounds: Focus on their mechanisms of action. Biochem. Pharmacol..

[19] Yang G., Zhao K., Ju Y., Mani S., Cao Q., Puukila S., Khaper N., Wu L., Wang R. (2013). Hydrogen sulfide protects against cellular senescence via S-sulfhydration of Keap1 and activation of Nrf2. Antioxid. Redox Signal..

[20] Li M., Hu W., Wang R., Li Z., Yu Y., Zhuo Y., Zhang Y., Wang Z., Qiu Y., Chen K. (2022). Sp1 S-Sulfhydration Induced by Hydrogen Sulfide Inhibits Inflammation via HDAC6/MyD88/NF-kappaB Signaling Pathway in Adjuvant-Induced Arthritis. Antioxidants.

[21] Powell C.R., Dillon K.M., Matson J.B. (2018). A review of hydrogen sulfide (H_2_S) donors: Chemistry and potential therapeutic applications. Biochem. Pharm..

[22] Calderone V., Martelli A., Testai L., Citi V., Breschi M.C. (2016). Using hydrogen sulfide to design and develop drugs. Expert Opin. Drug Discov..

[23] Piragine E., Calderone V. (2021). Pharmacological modulation of the hydrogen sulfide (H_2_S) system by dietary H_2_S-donors: A novel promising strategy in the prevention and treatment of type 2 diabetes mellitus. Phytother. Res..

[24] Piragine E., Citi V., Lawson K., Calderone V., Martelli A. (2022). Potential Effects of Natural H_2_S-Donors in Hypertension Management. Biomolecules.

[25] Citi V., Martelli A., Brancaleone V., Brogi S., Gojon G., Montanaro R., Morales G., Testai L., Calderone V. (2020). Anti-inflammatory and antiviral roles of hydrogen sulfide: Rationale for considering H_2_S-donors in COVID-19 therapy. Br. J. Pharmacol..

[26] Wu D., Hu Q., Zhu Y. (2016). Therapeutic application of hydrogen sulfide donors: The potential and challenges. Front. Med..

[27] Cristea M., Noja G.G., Stefea P., Sala A.L. (2020). The Impact of Population Aging and Public Health Support on EU Labor Markets. Int. J. Environ. Res. Public Health.

[28] Brancaleone V., Roviezzo F., Vellecco V., De Gruttola L., Bucci M., Cirino G. (2008). Biosynthesis of H2S is impaired in non-obese diabetic (NOD) mice. Br. J. Pharmacol..

[29] Peh M.T., Anwar A.B., Ng D.S., Atan M.S., Kumar S.D., Moore P.K. (2014). Effect of feeding a high fat diet on hydrogen sulfide (H2S) metabolism in the mouse. Nitric Oxide.

[30] Ahmad F.U., Sattar M.A., Rathore H.A., Tan Y.C., Akhtar S., Jin O.H., Pei Y.P., Abdullah N.A., Johns E.J. (2014). Hydrogen sulphide and tempol treatments improve the blood pressure and renal excretory responses in spontaneously hypertensive rats. Ren. Fail..

[31] Bhatia M. (2012). Role of hydrogen sulfide in the pathology of inflammation. Scientifica.

[32] Gaddam R.R., Chambers S., Murdoch D., Shaw G., Bhatia M. (2017). Circulating levels of hydrogen sulfide and substance P in patients with sepsis. J. Infect..

[33] Košir M., Podbregar M. (2017). Advances in the Diagnosis of Sepsis: Hydrogen Sulfide as a Prognostic Marker of Septic Shock Severity. Ejifcc.

[34] Zhang H., Huang Y., Chen S., Tang C., Wang G., Du J., Jin H. (2021). Hydrogen sulfide regulates insulin secretion and insulin resistance in diabetes mellitus, a new promising target for diabetes mellitus treatment? A review. J. Adv. Res..

[35] Meng G., Ma Y., Xie L., Ferro A., Ji Y. (2015). Emerging role of hydrogen sulfide in hypertension and related cardiovascular diseases. Br. J. Pharmacol..

[36] Ngowi E.E., Sarfraz M., Afzal A., Khan N.H., Khattak S., Zhang X., Li T., Duan S.F., Ji X.Y., Wu D.D. (2020). Roles of Hydrogen Sulfide Donors in Common Kidney Diseases. Front Pharm..

[37] Zheng D., Chen Z., Chen J., Zhuang X., Feng J., Li J. (2016). Exogenous hydrogen sulfide exerts proliferation, anti-apoptosis, migration effects and accelerates cell cycle progression in multiple myeloma cells via activating the Akt pathway. Oncol. Rep..

[38] Flint B., Tadi P. (2023). Physiology, Aging. StatPearls.

[39] Franceschi C., Garagnani P., Morsiani C., Conte M., Santoro A., Grignolio A., Monti D., Capri M., Salvioli S. (2018). The Continuum of Aging and Age-Related Diseases: Common Mechanisms but Different Rates. Front. Med..

[40] Hozo S.P., Djulbegovic B., Hozo I. (2005). Estimating the mean and variance from the median, range, and the size of a sample. BMC Med. Res. Methodol..

[41] Ali S.E., Farag M.A., Holvoet P., Hanafi R.S., Gad M.Z. (2016). A Comparative Metabolomics Approach Reveals Early Biomarkers for Metabolic Response to Acute Myocardial Infarction. Sci. Rep..

[42] Alyan A.K., Hanafi R.S., Gad M.Z. (2021). Point-of-care testing and optimization of sample treatment for fluorometric determination of hydrogen sulphide in plasma of cardiovascular patients. J. Adv. Res..

[43] Bahadoran Z., Jeddi S., Mirmiran P., Kashfi K., Azizi F., Ghasemi A. (2022). Association between serum hydrogen sulfide concentrations and dysglycemia: A population-based study. BMC Endocr. Disord..

[44] Chen Y.H., Yao W.Z., Geng B., Ding Y.L., Lu M., Zhao M.W., Tang C.S. (2005). Endogenous hydrogen sulfide in patients with COPD. Chest.

[45] Disbrow E., Stokes K.Y., Ledbetter C., Patterson J., Kelley R., Pardue S., Reekes T., Larmeu L., Batra V., Yuan S. (2021). Plasma hydrogen sulfide: A biomarker of Alzheimer’s disease and related dementias. Alzheimer’s Dement..

[46] Feng Y.L., Fan J.H., Lin X.J., Yang J.C., Cui Q.H., Tang X.J., Xu G.H., Geng B. (2017). Facilitating the measurement of circulatory hydrogen sulfide with fluorescence probe-coated microplates. Beijing Da Xue Xue Bao. Yi Xue Ban.

[47] Gao L., Xu Z., Yin Z., Chen K., Wang C., Zhang H. (2015). Association of hydrogen sulfide with alterations of monocyte chemokine receptors, CCR2 and CX3CR1 in patients with coronary artery disease. Inflamm. Res..

[48] Grabowska-Polanowska B., Skowron M., Miarka P., Pietrzycka A., Śliwka I. (2017). The application of chromatographic breath analysis in the search of volatile biomarkers of chronic kidney disease and coexisting type 2 diabetes mellitus. J. Chromatogr. B Anal. Technol. Biomed. Life Sci..

[49] Guo R., Wu Z., Jiang J., Liu C., Wu B., Li X., Li T., Mo H., He S., Li S. (2017). New mechanism of lipotoxicity in diabetic cardiomyopathy: Deficiency of Endogenous H_2_S Production and ER stress. Mech. Ageing Dev..

[50] Hao Y.M., He D.W., Gao Y., Fang L.N., Zhang P.P., Lu K., Lu R.Z., Li C. (2021). Association of Hydrogen Sulfide with Femoral Bone Mineral Density in Osteoporosis Patients: A Preliminary Study. Med. Sci. Monit..

[51] Jain S.K., Bull R., Rains J.L., Bass P.F., Levine S.N., Reddy S., McVie R., Bocchini J.A. (2010). Low levels of hydrogen sulfide in the blood of diabetes patients and streptozotocin-treated rats causes vascular inflammation?. Antioxid. Redox Signal..

[52] Jain S.K., Manna P., Micinski D., Lieblong B.J., Kahlon G., Morehead L., Hoeldtke R., Bass P.F., Levine S.N. (2013). In African American type 2 diabetic patients, is vitamin D deficiency associated with lower blood levels of hydrogen sulfide and cyclic adenosine monophosphate, and elevated oxidative stress?. Antioxid. Redox Signal..

[53] Kuang Q., Xue N., Chen J., Shen Z., Cui X., Fang Y., Ding X. (2018). Low Plasma Hydrogen Sulfide Is Associated with Impaired Renal Function and Cardiac Dysfunction. Am. J. Nephrol..

[54] Li H., Feng S.J., Zhang G.Z., Wang S.X. (2014). Correlation of lower concentrations of hydrogen sulfide with atherosclerosis in chronic hemodialysis patients with diabetic nephropathy. Blood Purif..

[55] Lin Z., Huang S., Yu H., Sun J., Huang P., Zhong Y. (2020). Analysis of Plasma Hydrogen Sulfide, Homocysteine, and L-Cysteine in Open-Angle Glaucoma Patients. J. Ocul. Pharmacol. Ther..

[56] Longchamp A., MacArthur M.R., Trocha K., Ganahl J., Mann C.G., Kip P., King W.W., Sharma G., Tao M., Mitchell S.J. (2021). Plasma Hydrogen Sulfide Is Positively Associated With Post-operative Survival in Patients Undergoing Surgical Revascularization. Front. Cardiovasc. Med..

[57] Pan X., Zhang Y., Tao S. (2015). Effects of Tai Chi exercise on blood pressure and plasma levels of nitric oxide, carbon monoxide and hydrogen sulfide in real-world patients with essential hypertension. Clin. Exp. Hypertens..

[58] Perna A.F., Luciano M.G., Ingrosso D., Pulzella P., Sepe I., Lanza D., Violetti E., Capasso R., Lombardi C., De Santo N.G. (2009). Hydrogen sulphide-generating pathways in haemodialysis patients: A study on relevant metabolites and transcriptional regulation of genes encoding for key enzymes. Nephrol. Dial. Transplant..

[59] Peter E.A., Shen X., Shah S.H., Pardue S., Glawe J.D., Zhang W.W., Reddy P., Akkus N.I., Varma J., Kevil C.G. (2013). Plasma free H2S levels are elevated in patients with cardiovascular disease. J. Am. Heart Assoc..

[60] Polhemus D.J., Li Z., Pattillo C.B., Gojon G., Gojon G., Giordano T., Krum H. (2015). A novel hydrogen sulfide prodrug, SG1002, promotes hydrogen sulfide and nitric oxide bioavailability in heart failure patients. Cardiovasc. Ther..

[61] Qiu X., Liu K., Xiao L., Jin S., Dong J., Teng X., Guo Q., Chen Y., Wu Y. (2018). Alpha-lipoic acid regulates the autophagy of vascular smooth muscle cells in diabetes by elevating hydrogen sulfide level. Biochim. Biophys. Acta. Mol. Basis Dis..

[62] Ran R., Du L., Zhang X., Chen X., Fang Y., Li Y., Tian H. (2014). Elevated hydrogen sulfide levels in vitreous body and plasma in patients with proliferative diabetic retinopathy. Retina.

[63] Saito J., Mackay A.J., Rossios C., Gibeon D., Macedo P., Sinharay R., Bhavsar P.K., Wedzicha J.A., Chung K.F. (2014). Sputum-to-serum hydrogen sulfide ratio in COPD. Thorax.

[64] Suzuki K., Sagara M., Aoki C., Tanaka S., Aso Y. (2017). Clinical Implication of Plasma Hydrogen Sulfide Levels in Japanese Patients with Type 2 Diabetes. Intern. Med..

[65] Wang C., Han J., Xiao L., Jin C.E., Li D.J., Yang Z. (2014). Role of hydrogen sulfide in portal hypertension and esophagogastric junction vascular disease. World J. Gastroenterol..

[66] Wang W., Feng S.J., Li H., Zhang X.D., Wang S.X. (2015). Correlation of Lower Concentrations of Hydrogen Sulfide with Activation of Protein Kinase CbetaII in Uremic Accelerated Atherosclerosis Patients. Chin. Med. J..

[67] Whiteman M., Gooding K.M., Whatmore J.L., Ball C.I., Mawson D., Skinner K., Tooke J.E., Shore A.C. (2010). Adiposity is a major determinant of plasma levels of the novel vasodilator hydrogen sulphide. Diabetologia.

[68] Wu L., Ishigaki Y., Hu Y., Sugimoto K., Zeng W., Harimoto T., Sun Y., He J., Suzuki T., Jiang X. (2020). H_2_S-activatable near-infrared afterglow luminescent probes for sensitive molecular imaging in vivo. Nat. Commun..

[69] Xiao L., Dong J.H., Teng X., Jin S., Xue H.M., Liu S.Y., Guo Q., Shen W., Ni X.C., Wu Y.M. (2018). Hydrogen sulfide improves endothelial dysfunction in hypertension by activating peroxisome proliferator-activated receptor delta/endothelial nitric oxide synthase signaling. J. Hypertens..

[70] Zheng M., Zeng Q., Shi X.Q., Zhao J., Tang C.S., Sun N.L., Geng B. (2011). Erythrocytic or serum hydrogen sulfide association with hypertension development in untreated essential hypertension. Chin. Med. J..

[71] Perng D.W., Chen P.K. (2017). The Relationship between Airway Inflammation and Exacerbation in Chronic Obstructive Pulmonary Disease. Tuberc. Respir. Dis..

[72] Sharif S., Van der Graaf Y., Cramer M.J., Kapelle L.J., de Borst G.J., Visseren F.L.J., Westerink J., SMART study group (2021). Low-grade inflammation as a risk factor for cardiovascular events and all-cause mortality in patients with type 2 diabetes. Cardiovasc. Diabetol..

[73] Yilmaz M.I., Carrero J.J., Axelsson J., Lindholm B., Stenvinkel P. (2007). Low-grade inflammation in chronic kidney disease patients before the start of renal replacement therapy: Sources and consequences. Clin. Nephrol..

[74] Zhang Z., Zhao L., Zhou X., Meng X., Zhou X. (2022). Role of inflammation, immunity, and oxidative stress in hypertension: New insights and potential therapeutic targets. Front Immunol.

[75] Roe K. (2021). An inflammation classification system using cytokine parameters. Scand. J. Immunol..

[76] Fang L., Moore X.L., Dart A.M., Wang L.M. (2015). Systemic inflammatory response following acute myocardial infarction. J. Geriatr. Cardiol..

[77] Ong S.B., Hernandez-Resendiz S., Crespo-Avilan G.E., Mukhametshina R.T., Kwek X.Y., Cabrera-Fuentes H.A., Hausenloy D.J. (2018). Inflammation following acute myocardial infarction: Multiple players, dynamic roles, and novel therapeutic opportunities. Pharmacol. Ther..

[78] Roxburgh C.S., McMillan D.C. (2014). Cancer and systemic inflammation: Treat the tumour and treat the host. Br. J. Cancer.

[79] Kostner A.H., Nielsen P.S., Georgsen J.B., Parner E.T., Nielsen M.B., Kersten C., Steiniche T. (2021). Systemic Inflammation Associates With a Myeloid Inflamed Tumor Microenvironment in Primary Resected Colon Cancer-May Cold Tumors Simply Be Too Hot?. Front. Immunol..

[80] King P.T. (2015). Inflammation in chronic obstructive pulmonary disease and its role in cardiovascular disease and lung cancer. Clin. Transl. Med..

[81] Fujimoto K., Yasuo M., Urushibata K., Hanaoka M., Koizumi T., Kubo K. (2005). Airway inflammation during stable and acutely exacerbated chronic obstructive pulmonary disease. Eur. Respir. J..

[82] Fox B., Schantz J.T., Haigh R., Wood M.E., Moore P.K., Viner N., Spencer J.P., Winyard P.G., Whiteman M. (2012). Inducible hydrogen sulfide synthesis in chondrocytes and mesenchymal progenitor cells: Is H_2_S a novel cytoprotective mediator in the inflamed joint?. J. Cell. Mol. Med..

[83] Gambari L., Grigolo B., Grassi F. (2019). Hydrogen Sulfide in Bone Tissue Regeneration and Repair: State of the Art and New Perspectives. Int. J. Mol. Sci..

[84] Karwi Q.G., Bice J.S., Baxter G.F. (2018). Pre- and postconditioning the heart with hydrogen sulfide (H_2_S) against ischemia/reperfusion injury in vivo: A systematic review and meta-analysis. Basic Res. Cardiol..

[85] Hellmich M.R., Szabo C. (2015). Hydrogen Sulfide and Cancer. Handb. Exp. Pharmacol..

[86] Sekiguchi F., Sekimoto T., Ogura A., Kawabata A. (2016). Endogenous Hydrogen Sulfide Enhances Cell Proliferation of Human Gastric Cancer AGS Cells. Biol. Pharm. Bull..

[87] Mao Z., Yang X., Mizutani S., Huang Y., Zhang Z., Shinmori H., Gao K., Yao J. (2020). Hydrogen Sulfide Mediates Tumor Cell Resistance to Thioredoxin Inhibitor. Front. Oncol..

[88] Ascenção K., Szabo C. (2022). Emerging roles of cystathionine β-synthase in various forms of cancer. Redox Biol..

[89] Sogutdelen E., Pacoli K., Juriasingani S., Akbari M., Gabril M., Sener A. (2020). Patterns of Expression of H_2_S-Producing Enzyme in Human Renal Cell Carcinoma Specimens: Potential Avenue for Future Therapeutics. In Vivo.

[90] Yang G., Wu L., Jiang B., Yang W., Qi J., Cao K., Meng Q., Mustafa A.K., Mu W., Zhang S. (2008). H2S as a physiologic vasorelaxant: Hypertension in mice with deletion of cystathionine gamma-lyase. Science.

[91] Szlęzak D., Hutsch T., Ufnal M., Wróbel M. (2022). Heart and kidney H_2_S production is reduced in hypertensive and older rats. Biochimie.

[92] Alsaeedi A., Welham S., Rose P., Zhu Y.Z. (2023). The Impact of Drugs on Hydrogen Sulfide Homeostasis in Mammals. Antioxidants.

[93] Wang C., Xu G., Wen Q., Peng X., Chen H., Zhang J., Xu S., Zhang C., Zhang M., Ma J. (2019). CBS promoter hypermethylation increases the risk of hypertension and stroke. Clinics.

[94] Ganguly P., Alam S.F. (2015). Role of homocysteine in the development of cardiovascular disease. Nutr. J..

[95] Karger A.B., Steffen B.T., Nomura S.O., Guan W., Garg P.K., Szklo M., Budoff M.J., Tsai M.Y. (2020). Association Between Homocysteine and Vascular Calcification Incidence, Prevalence, and Progression in the MESA Cohort. J. Am. Heart Assoc..

[96] Zhu C., Liu Q., Li X., Wei R., Ge T., Zheng X., Li B., Liu K., Cui R. (2022). Hydrogen sulfide: A new therapeutic target in vascular diseases. Front. Endocrinol..

[97] Lv B., Chen S., Tang C., Jin H., Du J., Huang Y. (2021). Hydrogen sulfide and vascular regulation—An update. J. Adv. Res..

[98] Spezzini J., Piragine E., d’Emmanuele di Villa Bianca R., Bucci M., Martelli A., Calderone V. (2023). Hydrogen sulfide and epigenetics: Novel insights into the cardiovascular effects of this gasotransmitter. Br. J. Pharmacol..

[99] Aminzadeh M.A., Vaziri N.D. (2012). Downregulation of the renal and hepatic hydrogen sulfide (H2S)-producing enzymes and capacity in chronic kidney disease. Nephrol. Dial. Transplant.

[100] Askari H., Seifi B., Kadkhodaee M., Sanadgol N., Elshiekh M., Ranjbaran M., Ahghari P. (2018). Protective effects of hydrogen sulfide on chronic kidney disease by reducing oxidative stress, inflammation and apoptosis. EXCLI J..

[101] Assmann T.S., Brondani L.A., Boucas A.P., Rheinheimer J., de Souza B.M., Canani L.H., Bauer A.C., Crispim D. (2016). Nitric oxide levels in patients with diabetes mellitus: A systematic review and meta-analysis. Nitric Oxide.

[102] Hua W., Chen Q., Gong F., Xie C., Zhou S., Gao L. (2013). Cardioprotection of H2S by downregulating iNOS and upregulating HO-1 expression in mice with CVB3-induced myocarditis. Life Sci..

[103] Yang R., Jia Q., Liu X.F., Wang Y.Y., Ma S.F. (2017). Effects of hydrogen sulfide on inducible nitric oxide synthase activity and expression of cardiomyocytes in diabetic rats. Mol. Med. Rep..

[104] Ciccone V., Piragine E., Gorica E., Citi V., Testai L., Pagnotta E., Matteo R., Pecchioni N., Montanaro R., Di Cesare Mannelli L. (2022). Anti-Inflammatory Effect of the Natural H_2_S-Donor Erucin in Vascular Endothelium. Int. J. Mol. Sci..

