# Supplementary material for: Circulating Levels of Hydrogen Sulfide (H2S) in Patients with Age-Related Diseases: A Systematic Review and Meta-Analysis
Source: Biomolecules. 2023 Jun 21;13(7):1023. doi: 10.3390/biom13071023 (PMC10376967; doi:10.3390/biom13071023)
Supplement: Supplementary file 1 [file biomolecules-13-01023-s001.zip › biomolecules-2451097-supplementary.pdf]

S1. Search strategy

Medline and CENTRAL:

(H_2S OR hydrogen sulfide OR hydrogen sulphide) AND (plasma OR plasma level* OR blood OR blood level* OR serum OR serum level* OR plasma concentration* OR blood concentration* OR serum concentration* OR concentration* OR availability OR bioavailability OR production) AND (ageing OR age-related disorder* OR age-related disease* OR hypertension OR blood pressure OR angina OR angina pectoris OR myocardial infarction OR heart failure OR coronary artery disease OR acute coronary syndrome OR chronic coronary syndrome OR arrythmias OR cardiovascular disorder* OR atherosclerosis OR hypercholesterolemia OR diabetes OR type 2 diabetes OR diabetes mellitus OR type 2 diabetes mellitus OR metabolic syndrome OR metabolic disorder* OR osteoporosis OR neurodegenerative disease* OR neurodegenerative disorder* OR dementia OR Alzheimer OR Alzheimer's disease OR Parkinson OR Parkinson's disease OR glaucoma OR asthma OR COPD OR chronic obstructive pulmonary disease OR nephropathy OR chronic kidney disease OR cancer OR decline)

Scopus:

(TITLE-ABS-KEY (H_2S OR (hydrogen AND sulfide) OR (hydrogen AND sulphide)) AND TITLE-ABS-KEY (plasma OR (plasma AND level*) OR blood OR (blood AND level*) OR serum OR (serum AND level*) OR (plasma AND concentration*) OR (blood AND concentration*) OR (serum AND concentration*) OR concentration* OR availability OR bioavailability OR production) AND TITLE-ABS-KEY (ageing OR (age-related AND disorder*) OR (age-related AND disease*) OR hypertension OR (blood AND pressure) OR angina OR (angina AND pectoris) OR (myocardial AND infarction) OR (heart AND failure) OR (coronary AND artery AND disease) OR (acute AND coronary AND syndrome) OR (chronic AND coronary AND syndrome) OR arrythmias OR (cardiovascular AND disorder*) OR atherosclerosis OR hypercholesterolemia OR diabetes OR (type AND 2 AND diabetes) OR (diabetes AND mellitus) OR (type AND 2 AND diabetes AND mellitus) OR (metabolic AND syndrome) OR (metabolic AND disorder*) OR osteoporosis OR (neurodegenerative AND disease*) OR (neurodegenerative AND disorder*) OR dementia OR Alzheimer OR (Alzheimer's AND disease) OR Parkinson OR (Parkinson's AND disease) OR glaucoma OR asthma OR COPD OR (chronic AND obstructive AND pulmonary AND disease) OR nephropathy OR (chronic AND kidney AND disease) OR cancer OR decline))

Embase:

#44. #9 AND #42 AND #43

#43. 'hydrogen sulfide'

#42. #10 OR #11 OR #12 OR #13 OR #14 OR #15 OR #16 OR

#17 OR #18 OR #19 OR #20 OR #21 OR #22 OR #23 OR

#24 OR #25 OR #26 OR #27 OR #28 OR #29 OR #30 OR

#31 OR #32 OR #33 OR #34 OR #35 OR #36 OR #37 OR

#38 OR #39 OR #40 OR #41

#41. 'deterioration'

#40. 'malignant neoplasm'

#39. 'chronic kidney failure'

#38. 'kidney disease'

#37. 'chronic obstructive lung disease'

#36. 'asthma'

#35. 'glaucoma'

#34. 'parkinson disease'

#33. 'alzheimer disease'

#32. 'dementia'

#31. neurodegenerative AND disorder*

#30. 'degenerative disease'

#29. 'osteoporosis'

#28. 'metabolic disorder'

#27. 'metabolic syndrome x'

#26. 'non insulin dependent diabetes mellitus'

#25. 'diabetes mellitus'

#24. 'hypercholesterolemia'

#23. 'atherosclerosis'

#22. 'cardiovascular disease'

#21. 'arrythmia'

#20. 'chronic coronary syndrome'

#19. 'acute coronary syndrome'

#18. 'coronary artery disease'

#17. 'heart failure'

#16. 'heart infarction'

#15. 'angina pectoris'

#14. 'blood pressure'

#13. 'hypertension'

#12. 'geriatric disorder'

#11. age AND related AND disease*

#10. 'aging'

#9. #1 OR #2 OR #3 OR #4 OR #5 OR #6 OR #7 OR #8

#8. 'production'

#7. 'bioavailability'

#6. 'availability'

#5. 'concentration (parameter)'

#4. 'serum'

#3. blood

#2. 'blood level'

#1. 'plasma'

Table S1. Characteristics of the included studies, details of H_2S levels measurement and main results. Symbols: ↑ indicates an increase in circulating levels of H_2S in patients with disease *vs* the control group, ↓ indicates a decrease in levels of H_2S while = indicates that there are no differences in circulating levels of H_2S between the two groups. List of abbreviations: ADRDs: Alzheimer's disease and related dementias; AE-COPD: acute exacerbation of chronic obstructive pulmonary disease; AMI: acute myocardial infarction; CAD: coronary artery disease; CHD: chronic haemodialysis; CKD: chronic kidney disease; COPD: chronic obstructive pulmonary disease; CRC: colorectal cancer; GC: gas chromatography;

HCC: hepatic cancer; HPLC: high performance liquid chromatography; LC-MS/MS: liquid chromatography-tandem mass spectrometry; NPDR: non-proliferative diabetic retinopathy; PAD: peripheral artery disease; PDR: non-proliferative diabetic retinopathy; STEMI: ST-elevation myocardial infarction; T2D: type 2 diabetes.

Study name	Study design	Study population	Controls	n° patients/ n° controls	Men, % (patients/ controls)	Mean age ± SD or range, years (patients/ controls)	Biological sample	Method of measurement	H_2S levels in patients with disease *vs* control	Included in the meta-analysis
Ali, 2016	Case-control	Patients with acute STEMI or unstable angina pectoris	Healthy patients	60/15	53.3/46.7	56.0 ± 12.9/ 57.0 ± 13.2	Serum	ELISA kit	↑	No
Alyan, 2019	Case-control	Patients with AMI (STEMI and non-STEMI)	Healthy subjects	75/50	81.3/82.0	51.4 ± 8.7/ 46.1 ± 10.7	Plasma	Fluorescent probe DNS-Az and H_2S Analyzer	↑	Yes
Bahadoran, 2022	Cross-sectional	Patients with T2D	Patients without T2D	111/511	53.2/46.6	56.1 ± 12.5/ 41.0 ± 12.6	Serum	Spectrophotometric method (Methylene blue)	↓	Yes
Chen, 2005	Case-control	Patients with stable COPD or AE-COPD	Healthy subjects	64/13	70.3/46.2	70.3 ± 8.6/ 58.7 ± 8.3	Serum	Sulfide-sensitive electrode	↑	No
Disbrow, 2021	Case-control	Patients with ADRDs	Patients without ADRDs	15/42	13.3/14.2	68.5 ± 5.93/ 67.5 ± 9.6	Plasma	HPLC and derivatization with monobromobimane	=	No
Feng, 2017	Case-control	Hypertensive patients	Healthy volunteers	30/22	-	-	Serum	Fluorescent probe C7Az	↓	Yes
Gao, 2015	Cross-sectional	Patients with CAD (i.e., stable angina pectoris, unstable angina pectoris, non-STEMI, and STEMI)	Healthy subjects	63/11	75.0/27.3	71.9 ± 5.1/ 68.7 ± 8.9	Plasma	Spectrophotometric method (Methylene blue)	↓	Yes

Grabowska-Polanowska, 2017_ CKD	Case-control	Patients with CKD	Healthy volunteers	10/10	-	48.5 (26-84)/ 36 (24-60)	Plasma	Spectrophotometric method (Methylene blue)	↑	Yes
Grabowska-Polanowska, 2017_ CKD+T2D	Case-control	Patients with CKD and T2D	Healthy volunteers	10/10	-	60 (35-85)/ 36 (24-60)	Plasma	Spectrophotometric method (Methylene blue)	↑	Yes
Guo, 2017	Case-control	Patients with diabetic cardiomyopathy	Healthy controls	32/-	75.0/-	-	Plasma	Sulphur ion-selective electrode	↓	No
Hao, 2021	Cross-sectional	Patients with osteopenia or osteoporosis	Patients with normal bone mineral density	25/75	48.0/77.3	64.1 ± 2.2/ 50.2 ± 1.1	Serum	ELISA kit	↑	No
Jain, 2010	Case-control	Patients with T2D	Normal volunteers	63/14	-	52.0 ± 3.0/ 54.0 ± 7.0	Blood	Spectrophotometric method (Methylene blue)	↓	No
Jain, 2013	Case-control	Patients with T2D	Healthy controls	76/36	-	47.2 ± 1.6/ 42.7 ± 3.0	Blood	Spectrophotometric method (Methylene blue)	↓	No
Kuang, 2018	Case-control	Patients with non-dialysis CKD	Healthy individuals	157/37	50.3/48.6	49.5 ± 14.8/ 50.9 ± 6.4	Plasma	Spectrophotometric method (Methylene blue)	↓	Yes
Li, 2014_CHD	Case-control	Patients with CHD	Normal control group	36/30	55.6/50.0	48.3 ± 11.5/-	Plasma	Sulfide sensitive electrode	↓	Yes
Li, 2014_ CHD+T2D	Case-control	Patients with CHD and diabetic nephropathy	Normal control group	32/30	53.1/50.0	47.8 ± 12.5/-	Plasma	Sulfide sensitive electrode	↓	Yes
Lin, 2020	Cross-sectional	Patients with ocular hypertension, normal tension glaucoma or primary open-angle glaucoma	Normal subjects	114/78	50.9/46.2	58.7 ± 12.1/ 60.5 ± 1.5	Plasma	Spectrophotometric method (Methylene blue)	↓	Yes

Longchamp, 2021	Case-control	Patients with vascular disease who underwent carotid endarterectomy	Healthy patients	115/20	63.0/65.0	69.0 ± 9.0/ 68.0 ± 2.3	Plasma	Lead acetate method	↓	Yes
Pan, 2015	Case-control	Patients with essential hypertension	Healthy volunteers	10/16	62.5/62.5	56.9 ± 4.0/ 55.5 ± 3.6	Plasma	Spectrophotometric method (Methylene blue)	↓	Yes
Perna, 2009	Case-control	Haemodialysis patients (end stage renal disease)	Healthy controls	65/31	-	63 (39-69)/ 53 (29-59)	Plasma	Spectrophotometric method (Methylene blue)	↓	Yes
Peter, 2013	Cross-sectional	Patients with any vascular disease (CAD or PAD)	Healthy volunteers	219/53	60.7/28.3	56.5 ± 8.5/ 53.0 ± 8.6	Plasma	Reversed phase HPLC	↑	Yes
Polhemus, 2015	Case-control	Patients with heart failure	Healthy subjects	12/8	100.0/100.0	57.2 ± 13.5/ 27.4 ± 1.7	Plasma	GC chemiluminescence	=	No
Qiu, 2018	Case-control	Patients with T2D	Patients without T2D	101/20	61.4/40.0	61.4 ± 14.2/ 61.9 ± 9.9	Serum	LC-MS/MS	↓	Yes
Ran, 2014_T2D	Case-control	Patients with T2D	Normal controls	25/30	-	61.9 ± 2.0/ 57.8 ± 1.8	Plasma	Spectrophotometric method (Methylene blue)	↓	Yes
Ran, 2014_ T2D+NPDR	Case-control	Patients with T2D and NPDR	Normal controls	25/30	-	62.7 ± 1.9/ 57.8 ± 1.8	Plasma	Spectrophotometric method (Methylene blue)	↑	Yes
Ran, 2014_ T2D+PDR	Case-control	Patients with T2D and PDR	Normal controls	25/30	-	62.2 ± 2.3/ 57.8 ± 1.8	Plasma	Spectrophotometric method (Methylene blue)	↑	Yes
Saito, 2014_ COPD	Cross-sectional	Patients with stable COPD	Healthy subjects	64/35	51.6/54.3	69.1 ± 8.6/ 51.3 ± 8.5	Serum	Sulfide sensitive electrode	↑	Yes

Saito, 2014_ AE-COPD	Cross-sectional	Patients with AE-COPD	Healthy subjects	29/35	62.1/54.3	74.4 ± 9.1/ 51.3 ± 8.5	Serum	Sulfide sensitive electrode	↓	Yes
Suzuki, 2017	Case-control	Patients with T2D	Patients without T2D	154/66	43.5/37.9	61.7 ± 13.6/ 52.5 ± 14.8	Plasma	Spectrophotometric method (Methylene blue)	↓	Yes
Wang, 2014	Case-control	Patients with cirrhosis-induced portal hypertension	Healthy individuals	200/100	70.0/50.0	44.0 ± 14.0/ 47.0 ± 13.0	Plasma	Spectrophotometric method (Methylene blue)	↓	Yes
Wang, 2015	Case-control	Patients with CHD, with or without uremic accelerated atherosclerosis	Healthy people	60/30	61.7/50.0	47.2 ± 12.1/-	Plasma	Sulfide sensitive electrode	↓	No
Whiteman, 2010_T2D	Case-control	Patients with T2D	Lean, healthy volunteers	11/11	100.0/100.0	61.0 ± 8.4/ 54.8 ± 16.5	Plasma	Spectrophotometric method (Methylene blue)	↓	Yes
Whiteman, 2010_ overweight	Case-control	Overweight patients	Lean, healthy volunteers	16/11	100.0/100.0	65.0 ± 6.1/ 54.8 ± 16.5	Plasma	Spectrophotometric method (Methylene blue)	↓	Yes
Wu, 2020_HCC	Case-control	Patients with HCC	Healthy volunteers	10/10	-	-	Blood	Luminescent probe	↑	Yes
Wu, 2020_CRC	Case-control	Patients with CRC	Healthy volunteers	10/10	-	-	Blood	Luminescent probe	↑	Yes
Xiao, 2018	Cross-sectional	Patients with hypertension	Normotensive patients	15/22	67.0/68.0	64.0 ± 11.6/ 59.0 ± 9.4	Plasma	LC-MS/MS	↓	Yes
Zheng, 2011	Case-control	Patients with essential hypertension	Normotensive patients	62/64	72.6/43.8	47.6 ± 9.2/ 46.7 ± 10.1	Serum	Sulfide sensitive electrode	↑	Yes
Zheng, 2016	Case-control	Patients with multiple myeloma	Healthy subjects	20/15	55.0/-	57 (37-70)/-	Plasma	ELISA kit	↑	No

Table S2. Risk of bias of case-control studies.

Study name	1. Were the groups comparable other than the presence of disease in cases or the absence of disease in controls?	2. Were controls and patients with disease matched appropriately?	3. Were the criteria for inclusion/ exclusion in the sample clearly defined?	4. Were the study subjects and the setting described in detail?	5. Was the sample size enough in both control and disease groups?	6. Was the sample frame taken from an appropriate population base so that it closely represented the target/reference population under investigation?	7. Were confounding factors identified?	8. Were strategies to deal with confoundi ng factors stated?	9. Were the outcomes measured in a valid and reliable way?	10. Was appropri ate statistical analysis used?	Total score
Ali, 2016	0	1	1	1	0	0	0	0	1	1	5/10
Alyan, 2019	1	0	0	1	1	0	0	0	0	1	4/10
Chen, 2005	0	1	0	1	0	1	0	0	1	1	5/10
Disbrow, 2021	1	1	0	1	0	0	0	0	1	1	5/10
Feng, 2017	0	1	0	1	1	0	0	0	0	1	4/10
Grabowska-Polanowska, 2017	0	0	0	1	0	1	0	0	0	1	3/10
Guo, 2017	0	1	1	1	0	0	0	0	1	1	5/10

Hao, 2021	0	1	1	1	1	0	0	1	1	1	7/10
Jain, 2010	0	1	0	0	0	0	0	0	1	1	3/10
Jain, 2013	0	1	1	1	1	0	0	0	1	1	6/10
Kuang, 2018	0	1	1	1	1	1	0	0	1	1	7/10
Li, 2014	1	1	1	1	1	1	1	1	1	1	10/10
Longchamp, 2021	0	1	0	1	1	0	0	0	0	1	4/10
Pan, 2015	0	1	1	1	0	1	0	0	1	1	6/10
Perna, 2009	0	0	1	1	1	1	1	1	1	1	8/10
Polhemus, 2015	0	0	1	1	0	0	0	0	1	1	4/10
Qiu, 2018	1	1	1	1	1	1	1	1	1	1	10/10
Ran, 2014	1	0	0	1	1	1	0	0	1	1	6/10

Suzuki, 2017	0	0	1	1	1	1	0	0	1	1	5/10
Wang, 2014	0	0	1	1	1	1	1	1	1	1	8/10
Wang, 2015	0	1	1	1	1	1	0	0	1	1	7/10
Whiteman, 2010	0	1	1	1	0	0	0	0	1	1	5/10
Wu, 2020	0	0	0	1	0	0	0	0	0	1	2/10
Zheng, 2011	0	1	1	1	1	1	0	0	1	1	7/10
Zheng, 2016	0	1	0	1	0	0	0	0	1	1	4/10

Table S3. Risk of bias of cross-sectional studies.

Study name	1. Were the criteria for inclusion/ exclusion in the sample clearly defined?	2. Were the study subjects and the setting described in detail?	3. Was the sample size enough in both control and disease groups?	4. Were objective, standard criteria used for measurement of the condition (disease)?	5. Were confounding factors identified?	6. Were strategies to deal with confounding factors stated?	7. Were the outcomes measured in a valid and reliable way?	8. Was appropriate statistical analysis used?	Total score
Bahadoran, 2022	1	1	1	1	0	0	1	1	6/8
Gao, 2015	1	1	0	1	0	0	1	1	5/8
Hao, 2021	1	1	1	0	0	1	1	1	6/8
Lin, 2020	1	1	1	1	1	1	1	1	8/8
Peter, 2013	1	1	1	1	0	0	0	1	5/8
Saito, 2014	1	1	1	1	0	0	1	1	6/8
Xiao, 2018	0	1	0	1	0	0	1	1	4/8

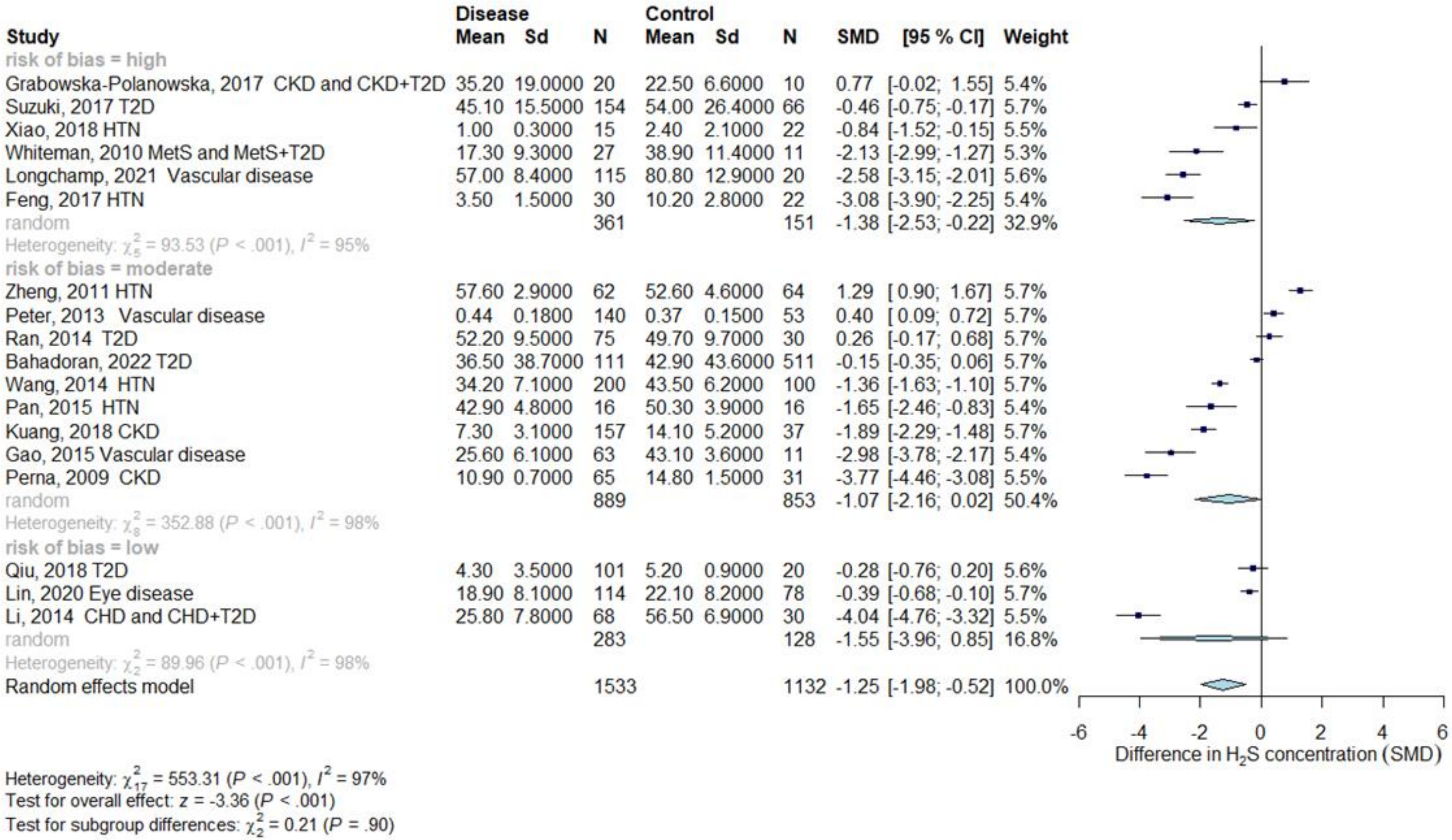

Figure S1. Forest plot with standardized mean differences (SMD) of circulating levels of H_2S, stratified by risk of bias, in patients with chronic age-related diseases characterized by a gradual decrease in organ and tissue functions and low-grade inflammation compared with subjects without disease (control group). Abbreviations: CHD, chronic haemodialysis; CKD: chronic kidney disease; HTN, hypertension; MetS, metabolic syndrome; T2D: type 2 diabetes.

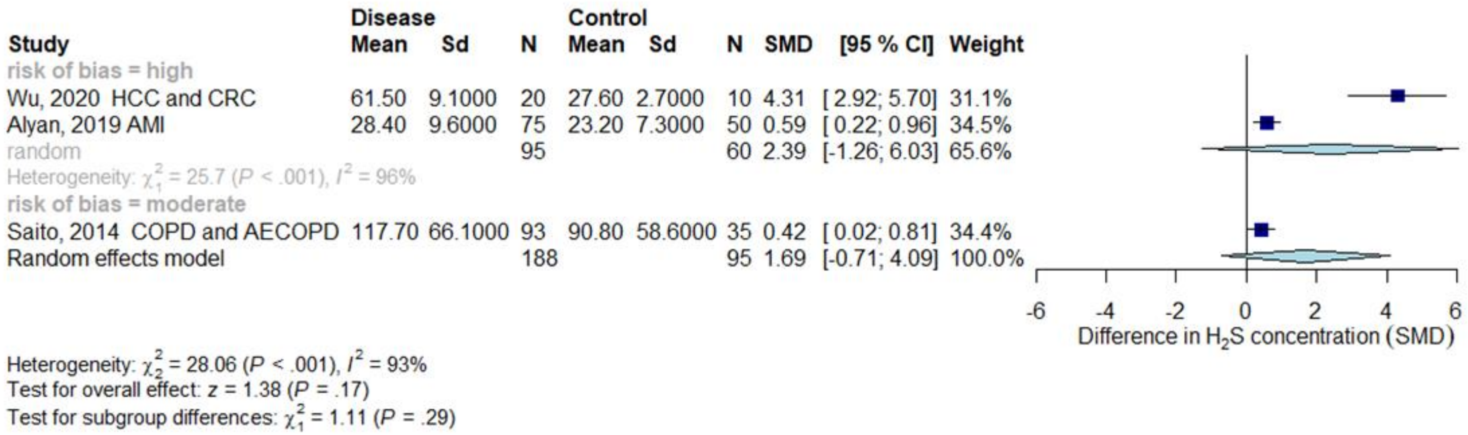

Figure S2. Forest plot with standardized mean differences (SMD) of circulating levels of H_2S, stratified by risk of bias, in patients with age-related diseases characterized by acute inflammation (e.g., AMI), acute exacerbations of disease (e.g., COPD) or singular inflammatory profile (e.g., cancer) compared with subjects without disease (control group). Abbreviations: AE-COPD, acute exacerbation of chronic obstructive pulmonary disease; AMI: acute myocardial infarction; COPD, chronic obstructive pulmonary disease; CRC, colorectal cancer; HCC, hepatic cancer.
